# New Insights into the Magnetic Properties of CoFe_2_O_4_@SiO_2_@Au Magnetoplasmonic Nanoparticles

**DOI:** 10.3390/nano12060942

**Published:** 2022-03-12

**Authors:** Rareș Bortnic, Adam Szatmari, Gabriela Souca, Răzvan Hirian, Roxana Dudric, Lucian Barbu-Tudoran, Valentin Toma, Rareș Știufiuc, Romulus Tetean, Emil Burzo

**Affiliations:** 1 Faculty of Physics, “Babes Bolyai” University, 400084 Cluj-Napoca, Romania; rares.bortnic@ubbcluj.ro (R.B.); adam.szatmari@ubbcluj.ro (A.S.); gabriela.souca@ubbcluj.ro (G.S.); razvan.hirian@ubbcluj.ro (R.H.); roxana.dudric@ubbcluj.ro (R.D.); emil.burzo@ubbcluj.ro (E.B.); 2 Electron Microscopy Center “Prof. C. Crăciun”, Faculty of Biology & Geology, “Babes-Bolyai” University, 400006 Cluj-Napoca, Romania; lucian.barbu@itim-cj.ro; 3 Integrated Electron Microscopy Laboratory, National Institute for Research and Development of Isotopic and Molecular Technologies, 400293 Cluj-Napoca, Romania; 4 Department of Bionanoscopy, MedFuture Research Center for Advance Medicine, “Iuliu Hatieganu” University of Medicine and Pharmacy, 400337 Cluj-Napoca, Romania; valentin.toma@umfcluj.ro (V.T.); rares.stiufiuc@umfcluj.ro (R.Ș.)

**Keywords:** core–shell nanoparticles, magnetoplasmonic nanoparticles, magnetic properties, spin-glass, exchange field

## Abstract

We report the successful synthesis and a complete magnetic characterization of CoFe_2_O_4_@SiO_2_@Au magnetoplasmonic nanoparticles. The CoFe_2_O_4_ magnetic nanoparticles were prepared using the hydrothermal method. A subsequent SiO_2_ shell followed by a plasmonic Au shell were deposited on the magnetic core creating magnetoplasmonic nanoparticles with a core–shell architecture. A spin-glass-type magnetism was shown at the surface of the CoFe_2_O_4_ nanograins. Depending on the external magnetic field, two types of spin-glass were identified and analyzed in correlation with the exchange field acting on octahedral and tetrahedral iron sites. The magnetization per formula unit of the CoFe_2_O_4_ core is not changed in the case of CoFe_2_O_4_@SiO_2_@Au nanocomposites. The gold nanoparticles creating the plasmonic shell show a giant diamagnetic susceptibility, dependent on their crystallite sizes.

## 1. Introduction

The flexibility of spinel ferrite CoFe_2_O_4_ or a (Co_1−x_Fe_x_)_T_(Co_x_Fe_2−x_)_O_O_4_ structure, with a variable occupancy of tetrahedral (T) and octahedral sites (O), provides a wide range of physical properties and applications, particularly in their nanosized form. As a function of the inversion parameter, x, the structure changes from a normal spinel (x = 0) to an inverse spinel type (x = 1). In a bulk state, the CoFe_2_O_4_ ferrite has mainly an inverse-type spinel structure and crystallizes in an FCC-type lattice, space group $Fm\bar{3}m$. The ferrite is ferrimagnetically ordered, and the magnetic moments of the atoms situated in octahedral and tetrahedral sublattices, respectively, being antiparallelly aligned. According to the degree of inversion, a large range of magnetizations can be obtained. The superexchange parameters inside and between magnetic sublattices were determined in the bulk state, starting from a mean field model [1,2].

At the nanometer scale, the magnetic behavior of CoFe_2_O_4_ ferrite shows significant differences with respect to that of the bulk state. The crossover to a single domain behavior is 40 nm [3]. The system becomes superparamagnetic between 7 and 10 nm. A core–shell model was proposed for CoFe_2_O_4_ nanograins, in which a core of aligned spins is surrounded by a magnetically disordered shell [4]. The low-temperature magnetic behavior of ultra-small CoFe_2_O_4_ nanoparticles was also associated with a random freezing of surface spins [5,6]. Thus, associated with size reduction and the formation of single-domain particles, the presence of superparamagnetism, a canted spin structure and surface anisotropy can be present, with different potential applications such as biomedical, electrical, antibacterial, energy storage media, coatings and magnetic refrigerants [7,8,9,10]. 

The CoFe_2_O_4_ nanoparticles are difficult to disperse due to their strong magnetic properties. A method to suppress nanoparticles’ agglomeration consists in surface modification by coating, leading to the creation of so-called core–shell nanocomposites. For medical applications, the coating shell must be non-toxic, biocompatible and stable in physiological environments. A silica shell protects and stabilizes the magnetic core, in addition to having low cytotoxicity, good chemical inertness and high thermal stability [9,10,11,12]. Additionally, it can be functionalized to bind on its surface molecules that possess silanol groups. 

The magnetoplasmonic core–shell nanocomposites, having a noble metal shell, are promising materials for biomedical applications. Metallic Au and Ag have a good surface-enhanced Raman scattering effect, whose surface plasma resonance enhances the electromagnetic field near the surface [13,14,15,16,17]. In this way the Raman scattering signals of the adsorbed molecules are greatly enhanced as compared with that of the ordinary Raman molecules. The gold does not interact with biological systems, so the use of Au on the shell surface is useful in supplying biocompatibility characteristics to nanostructures. 

The present paper reports the successful synthesis and physical characterization of CoFe_2_O_4_@SiO_2_@Au magnetoplasmonic nanoparticles. The CoFe_2_O_4_ magnetic nanoparticles have been synthesized by the hydrothermal method. Subsequently, the SiO_2_ shell was deposited on CoFe_2_O_4_ core nanoparticles. The further deposition of gold was made after the functionalization of CoFe_2_O_4_@SiO_2_ nanocomposites. Once the synthesis process was completed, a complete evaluation of their magnetic properties has been performed. A spin-glass-type magnetism was identified at the surface of CoFe_2_O_4_ nanograins. The magnetization per formula unit of CoFe_2_O_4_ core was not changed in the CoFe_2_O_4_@SiO_2_@Au nanocomposites, while the coercive fields decreased in the case of magneto-plasmonic nanohybrids. The gold nanoparticles on the shell showed a giant diamagnetic susceptibility, dependent on their crystallite sizes. 

## 2. Materials and Methods

### 2.1. Samples Preparation

The CoFe_2_O_4_ nanoparticles were prepared using a typical hydrothermal method [18,19]. The Fe(III)acetylacetonate (0.4 mmol) and Co(II) acetylacetonate (0.2 mmol) were dissolved in 55 mL ethylene glycol and kept at a temperature of 50 °C. Following this, 0.8 g of polyvinylpyrrolidone (PVP) was added to the solution. After magnetic stirring, the above solution was transferred to an autoclave, and heated to 240 °C at a rate of 0.3 °C/min and maintained at this temperature for 12 h. After cooling, the resulting product was washed several times using a 4/1 volumetric ratio of diethyl ether and ethanol. The CoFe_2_O_4_ nanoparticles were obtained by drying the solution at 50 °C. 

In order to deposit the SiO_2_ shell on CoFe_2_O_4_ nanoparticles, the Stober method was employed. The CoFe_2_O_4_ nanoparticles (0.2 mmol) were dispersed in 80 mL ethanol by ultrasonication and mechanical stirring; the dispersed particles were functionalized using 0.2 mL APTES. After ultrasonication and mechanical stirring, the shell deposition was completed during further sonication by adding TEOS (0.2 mL) to the solution and by increasing the pH upon the addition of the NH_4_OH solution. The solution was sonicated and stirred for 2 h, keeping the temperature constant at *t* = 40 °C. The nanocomposites thus obtained were washed several times with ethanol and then dispersed in water.

The CoFe_2_O_4_@SiO_2_ nanostructures were then functionalized with 0.2 mL of 3-aminopropyltriethoxysilane (APTES.) The obtained CoFe_2_O_4_@SiO_2_ particles were dispersed in water. The pH of the solution was lowered to 4 using a 1M HCl solution. Aqueous solutions of chloroauric acid (20 mmol/L), sodium citrate dehydrate(100 mmol/L), mixed trisodium citrate dehydrate (20 mmol/L) and sodium borohydride (50 mmol/L) were added sequentially and dropwise during sonification and mechanical stirring. The temperature was kept at 65 °C. Three series were prepared, denoted CoFe_2_O_4_@SiO_2_@AuN (N = 1–3). Finally, the nanocomposites were then washed.

### 2.2. Characterization

The morphology of the CoFe_2_O_4_ nanoparticles and of the CoFe_2_O_4_@SiO_2_@AuN nanocomposites was investigated by transmission electron microscopy (TEM) and scanning electron microscopy (SEM) using a Hitachi HD2700. The EDS measurements were done in order to analyze the composition of the prepared nanocomposites.

The crystal structure and crystallite sizes of CoFe_2_O_4_ nanoparticles and of the CoFe_2_O_4_@SiO_2_@AuN nanocomposites were determined by XRD measurements, performed at ambient temperature, with a Bruker D8 Advance diffractometer. The crystallite sizes were estimated by Rietveld refinement of XRD patterns, using FullProf Suite software.

Magnetic measurements were performed in the 4.2–300 K temperature range, and in external magnetic fields of up to 12 T, using a vibrating sample magnetometer from Cryogenic Limited (London, UK).

## 3. Results

### 3.1. Morphology and Crystal Structure

**Figure 1:**
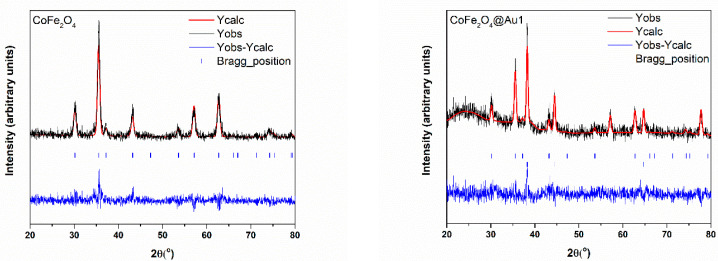

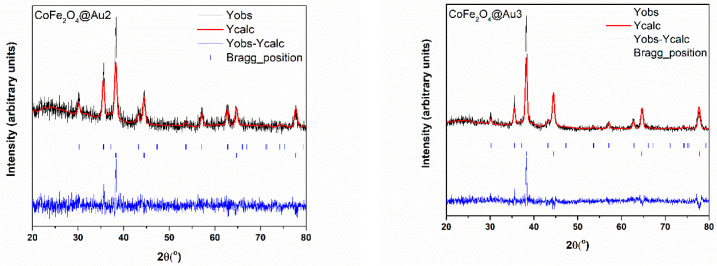
XRD patterns of CoFe_2_O_4_ and CoFe_2_O_4_@SiO_2_@Au nanocomposites and the results of Rietveld refinements.

The XRD patterns of CoFe_2_O_4_ and CoFe_2_O_4_@SiO_2_@AuN nanocomposites are shown in Figure 1. As expected, they correspond to the superposition of the XRD lines of CoFe_2_O_4_ and Au (ICDD PDF: 22-1086 for CoFe_2_O_4_ and 98-005-0876 for Au). All diffraction peaks including (111), (220), (311), (400), (422), (511) and (440) are fully indexed to the spinel CoFe_2_O_4_ phase. The sharp peaks corresponding to the (111), (200), (220) and (311) planes shown in the CoFe_2_O_4_@SiO_2_@AuN nanocomposite patterns confirm the crystallization of cubic FCC Au. The SiO_2_ was in amorphous state, as evidenced in XRD patterns by a broad feature at low angles. The lattice constants of CoFe_2_O_4_ and Au, determined from Rietveld refinements, are not dependent on nanostructures compositions and close to those of bulk samples. The crystallite sizes of CoFe_2_O_4_ nanoparticles are in the 14.2 nm and 20.2 nm range, while those of the gold nanoparticles were found to be between 15.9 nm and 23.6 nm (Table 1).

**Figure 2:**
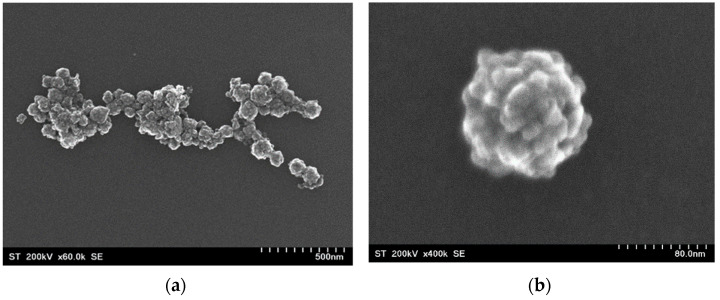

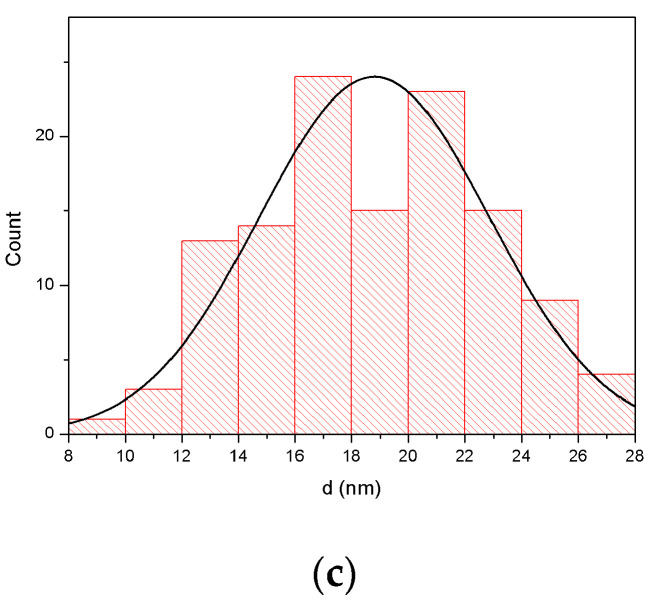
SEM images of CoFe_2_O_4_ nanoparticles (**a**,**b**) and particle size histogram (**c**).

The TEM and SEM images of CoFe_2_O_4_ nanoparticles evidenced that these were of nearly spherical form and agglomerated in separate pseudo-spherical “raspberry”-like nanostructures, with an average particle size of approximately 79 nm (Figure 2a,b). The mean size of the crystallites was around 19 nm, as can be seen in Figure 2c.

**Figure 3:**
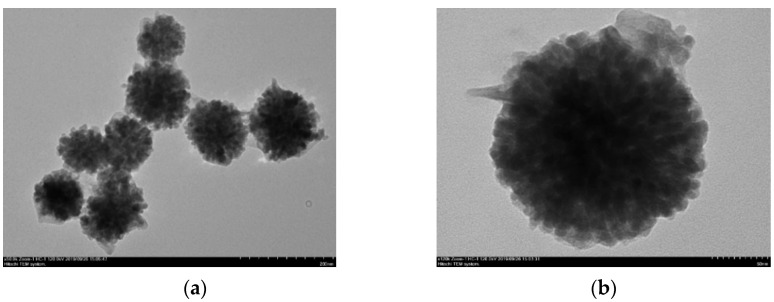
TEM images of CoFe_2_O_4_@SiO_2_ nanostructures (**a**) and of one isolated nanoparticle(**b**).

**Figure 4:**
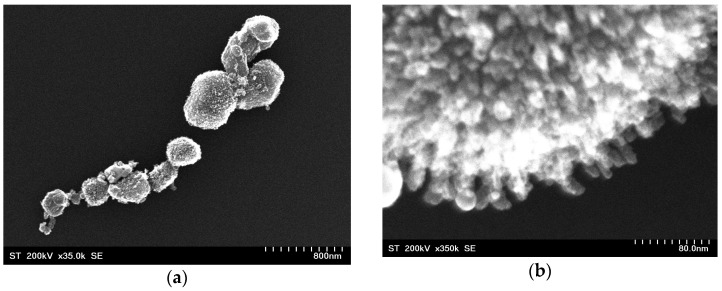
SEM images of CoFe_2_O_4_@SiO_2_@Au_2_ nanocomposite (**a**) and the edge of a nanoparticle.

After the deposition of SiO_2_, the TEM images show the formation of a shell layer (Figure 3). The nanocomposites thus formed had spherical or pseudo-spherical forms. A similar morphology was shown after gold deposition (Figure 4). The core–shell CoFe_2_O_4_@SiO_2_@AuN nanoparticles were nearly completely covered by gold. High resolution pictures confirmed that the Au nanoparticles had dimensions close to those determined by SEM, being in the 14–21 nm range. The average size of these nanocomposites was 288(57) nm. 

**Figure 5:**
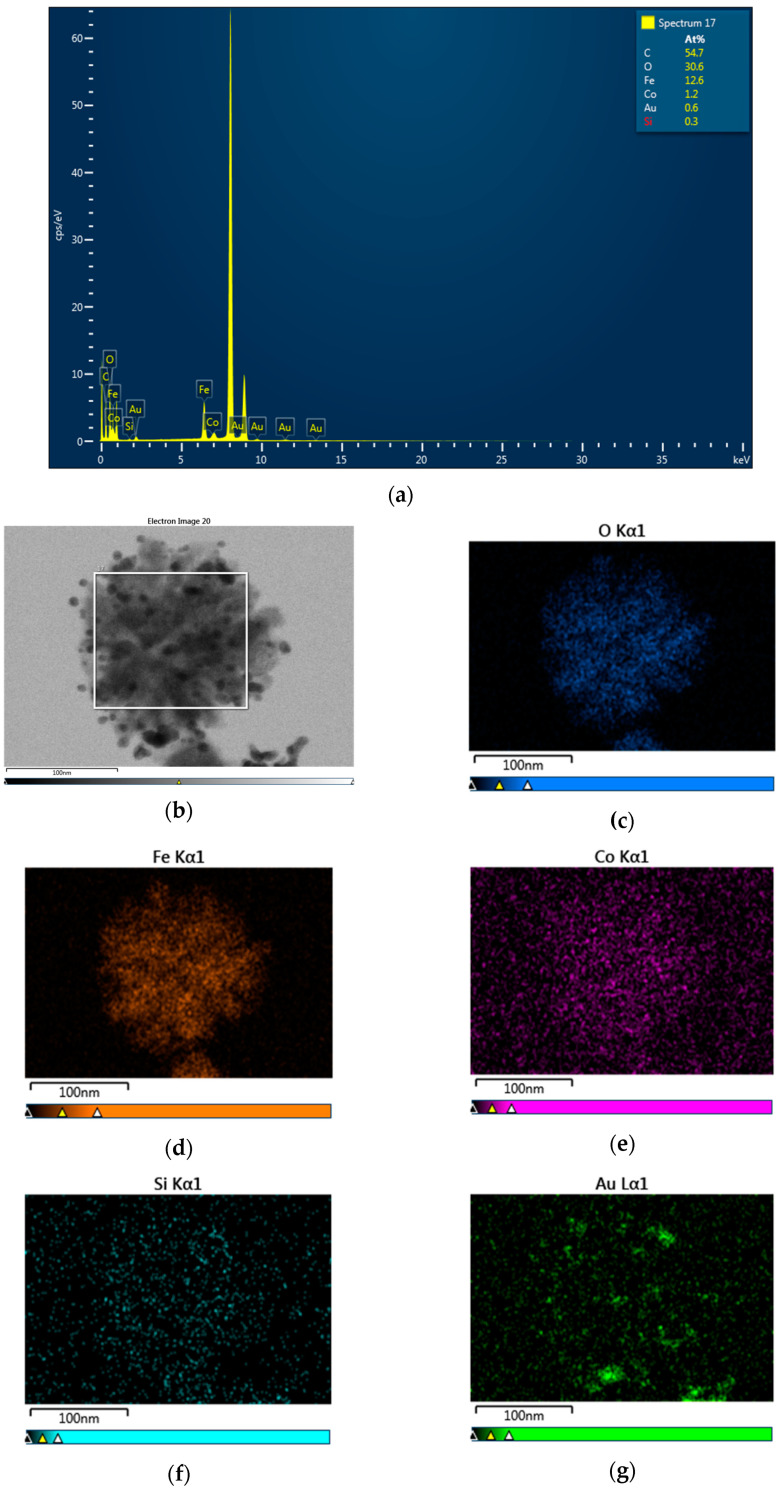
EDS spectrum (**a**), TEM image (**b**) EDS mapping (**c**–**g**) of CoFe_2_O_4_@SiO_2_@Au_2_ nanocomposite.

The EDS results show that the nanocomposites consisted of Fe, Co, O, Si and Au. As an example, the EDS results for CoFe_2_O_4_@SiO_2_@Au_2_ are given in Figure 5. The compositions determined for the studied samples are listed in Table 1.

### 3.2. Magnetic Properties

**Figure 6:**
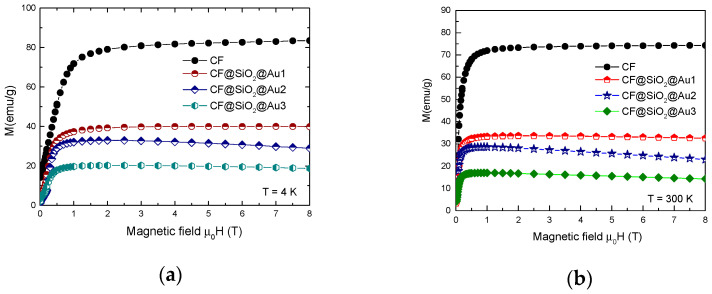
Magnetization isotherms at *T* = 4.2 K (**a**) and *T* = 300 K (**b**) of CoFe_2_O_4_ and CoFe_2_O_4_@SiO_2_@Au nanocomposites.

The magnetization isotherms, recorded at *T* = 4.2 K and 300 K for nanocrystalline CoFe_2_O_4_ and CoFe_2_O_4_@SiO_2_@AuN nanostructures, are shown in Figure 6. The presence of a spin-glass contribution superposed on mainly ferrimagnetic-type ordering is suggested in CoFe_2_O_4_ nanocrystalline samples by the present investigations. The spin canting could have been present due to the following: (1) surface effects due to symmetry breaking by the broken exchange bonds at the surface layer, (2) cation distribution in the tetrahedral and octahedral sites and (3) interactions between nanoparticles. In a spin-glass system, instead of having global anisotropy axes, there are easy axes whose directions vary randomly in space. Their direction is determined by the local spin arrangement. 

The previous Mössbauer studies [5,6] as well as infrared spectroscopy [4] performed on CoFe_2_O_4_ nanocrystalline samples evidenced the presence of a spin-glass state. It can be mentioned that in a large number of studies, ferrimagnetic-type behavior was also reported. The field dependences of the magnetization, in a spin-glass system, have been already analyzed [20]. In such a system, which shows high anisotropy (correlated spin glass), the approach to magnetic saturation follows a 1/*H*^2^ law. In systems with weak anisotropy (a ferromagnet with wondering axes) the approach to saturation when the external field, *H*, is smaller than the exchange field, *H*_ex_, can be described by a 1/*H*^1/2^ dependence, while for *H* > *H*_ex_ it follows the same dependence as for systems having high anisotropy. The spin-glass state of surface magnetization of weak anisotropic perovskites is well described by the 1/*H*^1/2^ law, as already reported [21,22,23]. 

**Figure 7:**
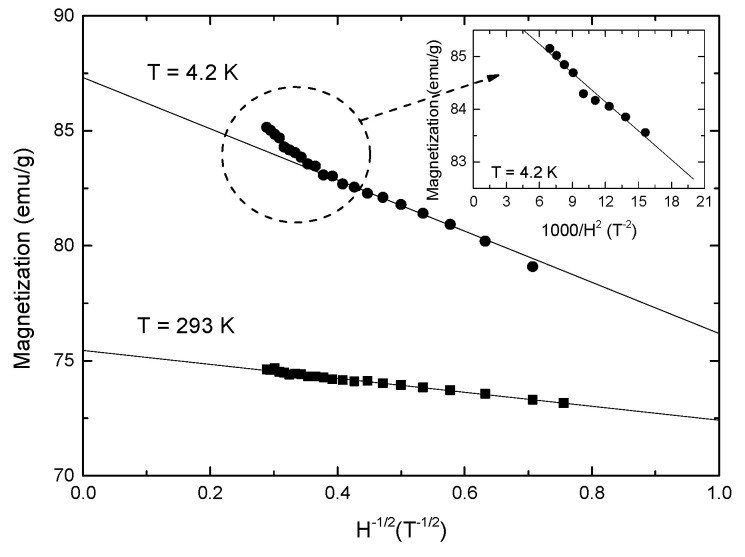
Magnetization isotherms of CoFe_2_O_4_ at *T* = 4.2 K and *T* = 300 K as function of *H*^1/2^. In inset, the data at *T* = 4.2 K for *H* > 8 T as a function of *H*^−2^.

The analysis of magnetization isotherms in CoFe_2_O_4_ nanograins suggests the presence of a spin-glass state superposed on mainly ferrimagnetic-type behavior. The dependences of magnetizations, at *T* = 4.2 K and 300 K on 1/*H*^1/2^, are given in Figure 7. Linear variations are present in the field ranges up to 12 T, for data obtained at *T* = 300 K and up to µ_0_*H* = 8 T at *T* = 4.2 K, described by the relation:

(1) $$\frac{M\left(H\right)}{M\left(0\right)}=1-bH^{{-}_{2}^{1}}$$

with a rate *b* = 0.1275 (T^1/2^) at *T* = 4.2 K and 0.0402 (T^1/2^) at *T* = 300 K. Nearly the same rate *b* has been obtained at *T* = 4.2 K for the field dependence of magnetization at the grain surface of Sr_2_FeMoO_6_-based perovskites [22,23].

For fields higher than 8–9 T, the magnetization at 4.2 K follows a 1/*H*^2^ law. There is a change in the shell magnetic behavior, suggesting a transition from a spin-glass state with wondering axes to a correlated spin-glass state. The extrapolation of magnetizations at *T* = 4.2 K, for both 1/*H*^1/2^ and 1/*H*^2^ trends, to infinite field, as expected, give the same value of saturation magnetization, *M*_s_ = 87.0 (3) emu/g. This corresponds to a magnetic moment per formula unit of *M*_s_ = 3.65 μ_B_/fu and it is expected to characterize the situation when the moments of both core and shell are oriented along the same axis. Thus, the inversion factor can be determined from saturation magnetization [24,25] as being x = (1/4)(7−*M*_s_) = 0.838. The composition of the sample (Co_0.162_Fe_0.838_)_T_(Co_0.838_Fe_1.162_)_O_O_4_ is closer to that of an inverse-spinel-type structure. 

The analysis of magnetization at *T* = 4.2 K suggests that the contribution of the spin-glass state to magnetization is around 8% of the total nanoparticle magnetization. This could be due to the cumulative effects of the broken exchange bonds at the surface layer, as well as to the exchange interactions between constituent ions, assuming a spherical grain with *d* = 20 nm and the shell volume having one CoFe_2_O_4_ lattice parameter (0.4 nm) width, corresponding to 11% of that of the nanograin. By Mössbauer spectroscopy, it was shown that the canting angle of iron moments in CoFe_2_O_4_ at tetrahedral sites was 41^0^ and at octahedral sites 36^0^ [5]. Thus, the corresponding magnetization of the shell volume, on the field direction, was ~8% of the total magnetization, suggesting that the spin-glass state is mainly due to the surface shell.

The exchange interactions at the level of the unit cell also influences the spin canting. The anisotropy of cobalt is sensitively higher than that of iron and no canting is expected for cobalt moments, unlike for the Fe^3+^ spins. The exchange fields acting on iron ions in octahedral and tetrahedral sites were estimated starting from the exchange interaction parameters in bulk CoFe_2_O_4,_ determined in the mean field model, assuming the presence of two [1] or three [2] magnetic sublattices. According to the determined inversion parameter, a tetrahedral Fe^3+^ ion has as neighbors four Fe and two Co octahedral ions, and an octahedral Fe^3+^ ion seven Co and five Fe tetrahedral ions, respectively [6]. In the above assumptions, the exchange fields, *H*_ex_, acting on iron at octahedral and tetrahedral sites are roughly of 300 T and 160 T, respectively. These values approximate those of the nanoparticle core. The exchange fields at the surface layer are sensitively diminished due to broken bonds and deviations from the parallel alignment of iron moments. Thus, for tetrahedral iron sites, in the one lattice parameter shell, the exchange field can be of a magnitude not highly different from the external field used for measurements. Consequently, there can be different magnetic responses of Fe^3+^ ions at tetrahedral and octahedral sites in the presence of an external field.

At ambient temperature, only a *T*^−1/2^ dependence of surface magnetization is shown, as determined by thermal effects.

**Figure 8:**
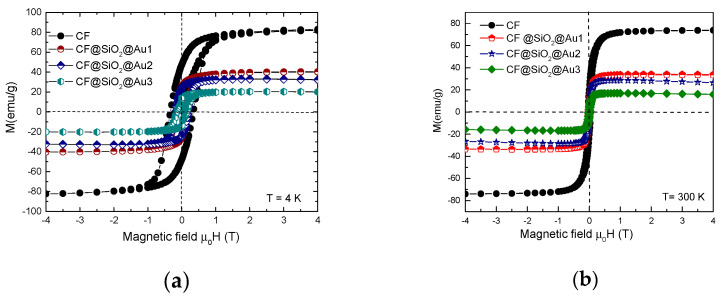
Hysteresis loops of CoFe_2_O_4_@SiO_2_@Au nanoparticles at *T* = 4.2 K (**a**) and *T* = 300 K (**b**).

The nanoparticle anisotropy seems to influence also the spin-canting-type behavior. The hysteresis curves recorded at 4.2 K and 300 K are given in Figure 8. At *T* = 4.2 K, the remanent magnetization was *M*_r_/*M*_s_ = 0.5, as expected for single domain particles. A smaller *M*_r_/*M*_s_ value was obtained at *T* = 300 K, correlated with the superparamagnetism of some nanoparticles having dimensions *d* < 10 nm—see Figure 8b. The anisotropy constants of CoFe_2_O_4_ nanoparticles were estimated assuming that below the blocking temperature, *T*_B_ the anisotropy is uniaxial [26]. According to the Stoner–Wohlfarth model for non-interacting single domain particles, the coercive field, *H*_c_, depends both on the anisotropy constant *K*_1_ and saturation magnetization [27]:

*H*_c_ = 2*K*_1_/µ_0_*M*_s_ (2)

From experimentally determined *H*_c_ values, anisotropy constants *K*_1_ = 7 × 10^5^ (*T* = 4.2 K) and 6.8 × 10^4^ (*T* = 300 K) J/m^3^ were obtained.

The effective anisotropy constant, *K*_eff_, has been estimated using the blocking temperature *T*_B_, according to the relation:

*T*_B_ = *K*_eff_*V*/25*k*_B_ (3)

where *V* is the nanoparticle volume.

For a log normal distribution of particles sizes, the blocking temperature, *T*_B_, is related to the temperature corresponding to the maximum in ZFC magnetization, *T*_max_, by the relation [28]: *T*_max_ = *e*<*T*_B_>, where *e* is in the 1.5–2.5 range. A careful analysis of the matter in case of CoFe_2_O_4_ nanoparticles with dimensions 5–7 nm evidenced a value *e* = 1.70 [29]. By using this value and taking into account that *T*_max_ = 300 K, an effective anisotropy constant *K*_eff_ = 4.31 × 10^4^ J/m^3^ was estimated assuming crystallite sizes of 15 nm. This value is close to that determined at *T* = 300 K from a coercive field. Somewhat higher *K*_eff_ values were obtained for nanograins with dimensions of 6.6 nm [16]. Starting from the determined anisotropy constants, the anisotropy field *H*_a_ = 2*K*/µ_0_*M* of 9–14 T was estimated. These values are smaller than the exchange field, but close to the field where a change in the spin-glass-type behavior was shown, suggesting possible relations.

The magnetization isotherms for CoFe_2_O_4_@SiO_2_@AuN nanostructures can be described by the contributions of a magnetic ordered phase, attributed to CoFe_2_O_4_ and diamagnetic contributions of SiO_2_ and Au—see Figure 6.

*M*_T_ = x*M*_CoFe2O4_ − (yχ_SiO2_ + zχ_Au_)*H* (4)

**Figure 9:**
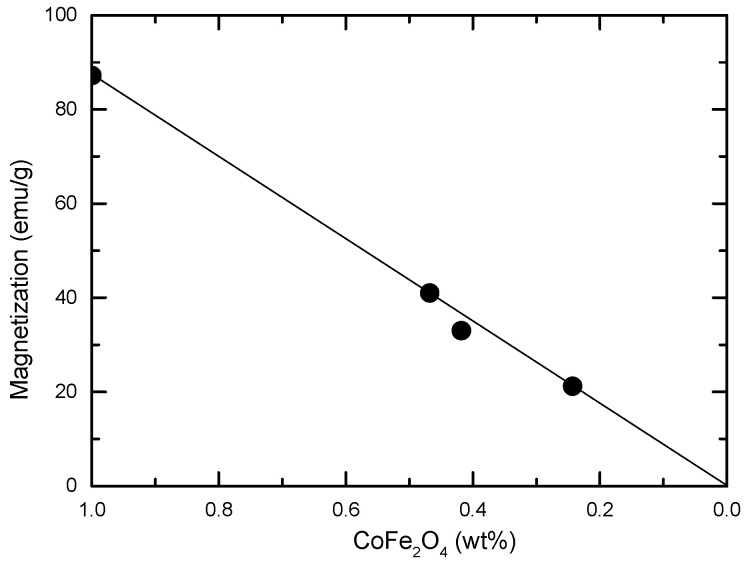
Magnetizations at *T* = 4.2 K as a function of CoFe_2_O_4_ content in CoFe_2_O_4_@SiO_2_@AuN nanocomposites. The solid line gives the expected trend in a simple dilution magnetic model.

By fitting the experimental data with the above relation, the contributions of CoFe_2_O_4_ to the nanostructure magnetizations were obtained. These values are in good agreement with the content of CoFe_2_O_4_ in the nanocomposites, as expected in the case of a simple magnetic dilution model—see Figure 9 and Table 1. The covering of the CoFe_2_O_4_ core with SiO_2_ and Au shells do not induce changes in their magnetic properties as compared with that of the single CoFe_2_O_4_ phase. Thus, by varying the CoFe_2_O_4_ content in nanostructures it is possible to ensure the desired magnetic properties of nanocomposites.

The diamagnetic contributions of the SiO_2_ and Au content, as determined from VSM studies, are higher than those expected starting from those of bulk SiO_2_ and Au, χ_SiO2_ = 0.447 × 10^−6^ [30] and χ_Au_ = 2.74 × 10^−6^ [31], respectively. Unlike that of Au, the diamagnetism of SiO_2_ is not affected by the nanocrystalline sizes. A giant diamagnetism has been observed already in gold nanorods [32] and theoretically investigated [33,34]. After the subtraction of the contribution of SiO_2_ to nanostructural diamagnetism, the diamagnetic susceptibility of gold nanoparticles was shown to be higher by one order of magnitude than that of the bulk value.

**Figure 10:**
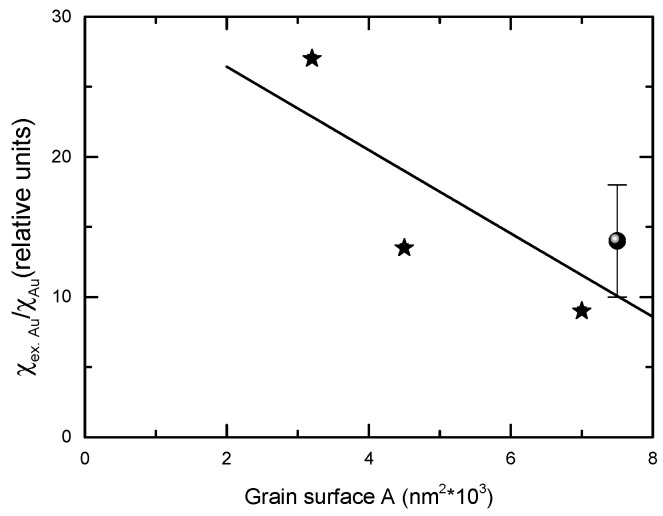
The gold diamagnetism in CoFe_2_O_4_@SiO_2_@AuN nanostructures as function of the mean surface of Au nanograins, in relative units (referred to bulk gold diamagnetism) (_*_). The data from [31] are also given (●).

The giant diamagnetic susceptibility of gold nanoparticles is a consequence of field-induced currents in the surface electrons [33]. The diamagnetic susceptibility is originated by steady currents induced by the applied field for quasi-free electrons confined in the surface. The diamagnetic response, induced when the external field is turned on, remains constant during the time the field is acting. As the size of the sample increases, the percentage of surface atoms decreases. Consequently, the magnetism of the surface approached that of the bulk sample. Such a trend is evidenced by the present data. The diamagnetism of gold nanoparticles, in relative units *s* = χ_ex.Au_/χ_Au_, is plotted in Figure 10, as a function of nanocrystallites surfaces, assuming that these have a spherical form. On the same figure, the *s* parameter determined for a nanorod with *d* = 15 nm and *l* = 80 nm is also given [32]. These data are in agreement with the expected trend between gold diamagnetism and nanoparticle dimensions

**Figure 11:**
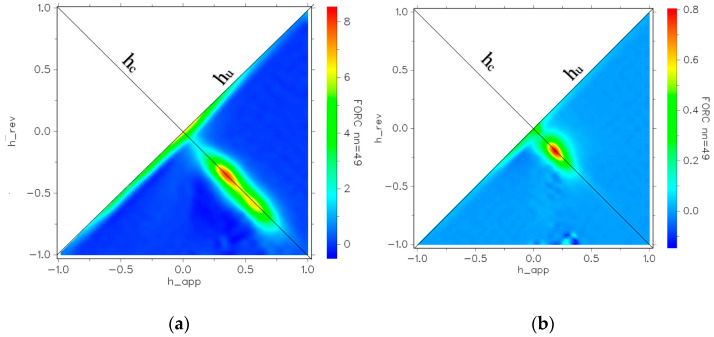
FORC diagrams for CoFe_2_O_4_ (**a**) and CoFe_2_O_4_@SiO_2_@Au2 (**b**) nanocomposites. h_rev denotes the applied field for each inversion curve. h_app is the applied field after that inversion is realized. nn is the smoothing factor.

The coercivities of CoFe_2_O_4_ and CoFe_2_O_4_@SiO_2_@AuN nanostructures determined from hysteresis loops, recorded at 4.2 K and 300 K, are given in Table 1. The coercive fields at *T* = 4.2 and 300 K increase due to reduction in CoFe_2_O_4_ nanosizes. At ambient temperatures, these values are smaller by one order of magnitude than those determined at *T* = 4.2 K. These magnetic measurements fail to fully describe complex magnetic nanostructures, such as an ensemble of nanoparticles with different magnetic properties, mainly due to the size distribution of the grains. The first-order reversal curve (FORC) diagram offers an image related to the coercivity and interaction fields acting on the different magnetic entities within the sample [35,36,37,38]. After applying a magnetic field to ensure the saturation magnetization, this is reduced to a predefined field *H*_r_, denoted as the reversal field, and the magnetization of the sample is measured while *H* is returned. The above sequence is repeated with a decreasing *H*_r_, thus obtaining a sequence of magnetization curves *M*(*H*_r_,*H*). On this basis, the FORC distribution, *P*, defined as a second derivative of the magnetization with respect to reversal and applied field, is obtained. The quantitative analysis of the FORC data is performed using the projection of the FORC distribution in the plane of the coercive field, *H*_c_ = (1/2)(*H* − *H*_r_), and the interaction field, *H*_u_ = (1/2)(*H* + *H*_r_), axes, called the coercivity distribution (*P*_Hc_) and interaction distribution (*P*_Hu_), respectively. The FORC distributions for the CoFe_2_O_4_ and CoFe_2_O_4_@SiO_2_@Au_2_ nanostructures are given in Figure 11. In CoFe_2_O_4_ nanograins, there is a large distribution of coercive fields and interaction fields.

The latter is due to particle interactions and the former is due to different particles switching at different applied field strengths. A higher maximum in the *p* value indicates stronger exchange interactions. The maximum of the probability density for CoFe_2_O_4_ is one order of magnitude larger than that for CoFe_2_O_4_@SiO_2_@AuN. This means that the FORC distribution of CoFe_2_O_4_ is much narrower than that for CoFe_2_O_4_@SiO_2_@AuN nanocomposites. The extended spot shown in the FORC diagram of CoFe_2_O_4_ resembles ‘’single phase” behavior, but with large exchange interactions between the nanograins along the h_c_-axis. In CoFe_2_O_4_@SiO_2_@Au2 nanostructures, the peak distribution is located at an interaction field of 0.19 T. The peak in the FORC distribution is thus shifted towards positive reversal and interaction fields in the core–shell nanostructures, dipolar interactions being increasingly more important due to the presence of a SiO_2_-Au shell and its screening effect.

## 4. Conclusions

Magnetoplasmonic CoFe_2_O_4_@SiO_2_@Au nanoparticles were successfully prepared. The SiO_2_ shell was mainly in an amorphous state, as evidenced in XRD patterns by a broad feature at low angles. The lattice constants of CoFe_2_O_4_ and Au were not dependent on nanostructure compositions being close to those of bulk samples. The crystallite sizes of CoFe_2_O_4_ nanoparticles were in the 14.2 nm and 20.2 nm range, while those of gold were between 15.9 nm and 23.6 nm. The TEM and SEM images of CoFe_2_O_4_ nanoparticles evidenced that these are of nearly spherical form and agglomerate in separate pseudo-spherical “raspberry”-like nanostructures. The presence of a spin-glass contribution superposed on mainly ferrimagnetic-type ordering is suggested. The contribution of the spin-glass state to magnetization was no more than 8% of the total CoFe_2_O_4_ nanoparticle magnetization. This could have been due to the cumulative effects of the broken exchange bonds at the surface layer, as well as to the exchange interactions between constituent ions. As a function of the external magnetic field, two types of spin-glasses were observed and correlated with different exchange fields acting on tetrahedral and octahedral iron sites.

The diamagnetic susceptibility of gold nanoparticles was shown to be by one order of magnitude higher than that of the bulk value. The giant diamagnetic susceptibility of gold nanoparticles is a consequence of field induced currents in the surface electrons. The diamagnetic susceptibility originated by the steady currents induced by the applied field for quasi-free electrons was confined in the surface. As the size of the sample increased, the percentage of surface atoms decreased. Consequently, the diamagnetism approached that of the bulk sample.

The FORC diagram of CoFe_2_O_4_ highlighted ‘’single phase” behavior, with large exchange interactions between the nanograins along the h_c_-axis. The peak in the FORC distribution was shifted towards positive reversal and interaction fields in the core–shell nanostructures, dipolar interactions increasing due to the presence of SiO_2_@Au shells and their screening effects.

## Figures and Tables

**Table 1:** Compositions, lattice parameters, crystallites sizes, magnetizations and coercive fields. AuN is a symbol for CoFe_2_O_4_@SiO_2_@AuN nanostructures with N = 1–3).

Nanostructure		CoFe_2_O_4_	Au1	Au2	Au3
Composition (weight %)	CoFe_2_O_4_	100	46.8	41.8	24.3
	SiO_2_	-	11.0	11.6	6.3
	Au	-	42.2	46.6	69.4
Lattice parameter (nm)	CoFe_2_O_4_ core	0.8379 (9)	0.8375 (4)	0.8372 (9)	0.8376 (9)
Crystallite size (nm		14.2 (2)	20.2 (3)	17.3 (2)	20.2 (2)
Lattice parameter (nm)	Au shell	-	0.4073 (2)	0.4073 (9)	0.4074 (7)
Crystallite size (nm		-	23.57 (2)	15.86 (4)	18.88 (6)
Nanocomposite magnetization/CoFe_2_O_4_ weight percent (emu/g)		86.9	86.1	84.5	85.5
Coercive field H_C_(T)	*T* = 4.2 k	0.35	0.20	0.28	0.19
	*T* = 300 k	0.04	0.03	0.03	0.030

## Data Availability

Not applicable.
